# Cytoscape App Store

**DOI:** 10.1093/bioinformatics/btt138

**Published:** 2013-04-16

**Authors:** Samad Lotia, Jason Montojo, Yue Dong, Gary D. Bader, Alexander R. Pico

**Affiliations:** ^1^Gladstone Institutes, San Francisco, CA 94158, USA and ^2^The Donnelly Centre, University of Toronto, Toronto, Ontario M5S 3E1, Canada

## Abstract

**Summary:** Cytoscape is an open source software tool for biological network visualization and analysis, which can be extended with independently developed apps. We launched the Cytoscape App Store to highlight the important features that apps add to Cytoscape, enable researchers to find and install apps they need and help developers promote their apps.

**Availability:** The App Store is available at http://apps.cytoscape.org.

**Contact:** apico@gladstone.ucsf.edu

## 1 INTRODUCTION

Cytoscape is an open source software tool for network visualization and analysis, primarily for biological data (Cline *et al.*, 2007; Shannon *et al.*, 2003). When users first install Cytoscape, it has a small well-defined set of functionality for working with networks. In addition, Cytoscape has an open architecture so that others can extend its functionality with apps. Cytoscape apps are additional software packages independently developed, licensed and installed. The wide-ranging functionality and specialization of Cytoscape principally reside in its apps. Over 150 free apps have been contributed to our Web site by developers from over a dozen countries.

Originally, the Cytoscape team maintained a catalog of submitted apps (called ‘plugins’ at the time) that were accessible from a web page or from within Cytoscape. The catalog had several limitations. Search was limited to exact matches to app names; users could not search by app description, category or author; and apps were categorized by single terms chosen by the Cytoscape team, not app developers. Each app entry in the catalog listed all versions of an app and did not emphasize the latest version. This catalog did not make it easy for users to discover apps in which they might be interested, e.g. based on their functionality. Moreover, when users did find an interesting app through the web page catalog, they had to search for the app again in Cytoscape to install it.

**Fig. 1.**
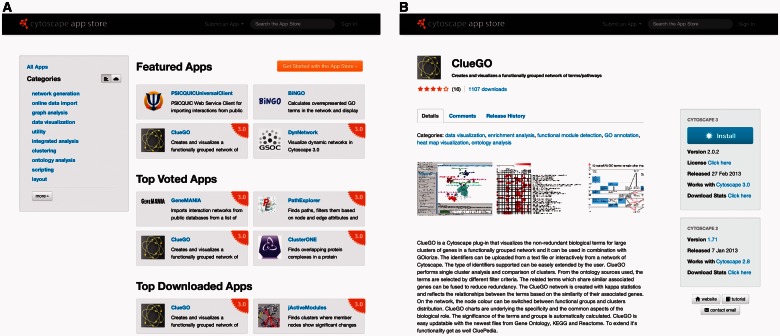
(**A**) The Cytoscape App Store main page, with three ways for discovering apps: Featured Apps, Categories and Search. (**B**) A Cytoscape app page, where users can learn about the app through screenshots, a description and tutorial and Web site links. Users can rate, review and perform one-click installation from their browser

To help address the needs of users, we launched the Cytoscape App Store (Fig. 1A) to coincide with the release of Cytoscape 3.0, a major re-architecturing of Cytoscape for improved stability, performance and versatility. The overarching goals of the Cytoscape App Store are to highlight the important features apps add to Cytoscape, to enable researchers to find apps they need and for developers to promote their apps. Here, we describe the features of the App Store for both users and app developers.

## 2 METHODS

The App Store offers multiple ways for users to discover apps (Fig. 1A). First, we provide a Featured Apps section that invites new users of Cytoscape to simply click on a featured app, read about it and, with a single click, install it. Thus, we promote a learning-by-action approach to understanding the role and scope of apps by maintaining a low barrier to entry. Second, there is a list of categories for users with a general idea of the kind of app in which they are interested. Users find all apps of a given type by clicking on a category. An app can belong to several categories, each chosen by their authors. Third, users can search for an app based on its name, description, categories, authors and authors’ institutions. With these features, we hope to encourage users to browse the App Store, discover and learn about apps, and install them to expand the capabilities of Cytoscape.

App pages have several aspects to help users learn about a particular app’s capabilities and usage (Fig. 1B). App authors can provide screenshots and in-depth descriptions on their app page. They can also provide links to their own Web site and tutorials. Though not required, we encourage authors to release their app under standard open source practices and to provide a link to a code repository. Users can also learn about the popularity of an app by its rating and number of downloads. Any visitor to an app page can rate it from zero to five stars, with five stars being the best. Each app has a download statistics page with a timeline plot of downloads per version and a world map showing where downloads occurred. These app statistics are often valuable to authors, as well as potential users, to track usage and justify further development and support of their work.

Apps can be installed right from the App Store with a single click without having to leave the browser when Cytoscape 3.0 or above is running. If an already installed app is out-of-date, it can be upgraded from the Web site using the same feature. The most recent version of an app is prominently displayed; older versions of an app can be manually downloaded in the Release History panel. From within Cytoscape, apps can also be searched for, installed, upgraded and uninstalled using the App Manager tool. The underlying goal of these features is to make app installation as easy as possible. We hope to entice users to install and experiment with apps by removing cumbersome and confusing steps.

Aside from users, app authors also benefit from the App Store. Authors can directly edit their app pages with their changes reflected in real-time preview. They can provide a custom icon, screenshots and a unique description to distinguish their app. Authors can submit new apps to the App Store with immediate technical feedback for ensuring that submissions follow Cytoscape’s metadata conventions. Authors can also post a contact email address and a link to their app's source code to encourage user engagement.

## 3 CONCLUSION

The App Store was designed with the latest Cytoscape 3.0 architecture in mind. For each Cytoscape 3.0 app, the App Store supports unique features like one-click install and comprehensive download statistics. 2.x Cytoscape apps are also available on the App Store despite being incompatible with Cytoscape 3.0. We plan to continue supporting these apps throughout the transition to Cytoscape 3.0, though we anticipate rapid growth in new and ported apps for 3.0 as it gains adoption in the community.

The App Store is already playing a broader role in the Cytoscape community than just a place for browsing and submitting apps. For instance, we held a competition for the best Cytoscape 3.0 apps in December 2012 (http://nrnb.org/competitions.html). The first prize was shared by ClueGO, which visualizes the relationship between gene ontology terms (Bindea *et al.*, 2009; http://apps.cytoscape.org/apps/cluego), and DynNetwork, which visualizes networks with time-based movement (http://apps.cytoscape.org/apps/dynnetwork). We plan to host more competitions in the future to encourage Cytoscape 3.0 app development.

Apps and the app developer community play a critical role in success of Cytoscape, ensuring its continued relevance and reach as the field of network biology evolves. The new Cytoscape App Store aims to increase the visibility and accessibility of apps, providing support to both Cytoscape users and app developers. We anticipate that traffic will continue to increase as apps—and the App Store—become more prominent in the Cytoscape community.

*Funding*: This work is supported by a grant from the Biomedical Technology, Bioinformatics and Computational Biology (BBCB) at National Institute of General Medical Sciences (NIGMS), National Institutes of Health (NIH) [P41 GM103504].

*Conflict of Interest*: none declared.

## References

[btt138-B1] Bindea G (2009). ClueGO: a Cytoscape plug-in to decipher functionally grouped gene ontology and pathway annotation networks. Bioinformatics.

[btt138-B5] Cline MS (2007). Integration of biological networks and gene expression data using Cytoscape. Nat. Protoc..

[btt138-B6] Shannon P (2003). Cytoscape: a software environment for integrated models of biomolecular interaction networks. Genome Res..

